# Influence of genetic variants on the response to long-acting β2-agonists (LABAs) and long-acting muscarinic antagonists (LAMAs) and combination therapies in patients with chronic obstructive pulmonary disease (COPD): a systematic review

**DOI:** 10.3389/fphar.2026.1973557

**Published:** 2026-10-29

**Authors:** Bárbara Torrecillas-Baena, Yolima Puentes-Osorio, Viviana Ramírez, Manuel Alejandro Salamanca-Miñarro, Andrea García-Alcaide, Luis S. M. Pajuelo

**Affiliations:** Duponte Investigación y Desarrollo S.L., Seville, Spain

**Keywords:** *ADRB2*, COPD, genetic variants, pharmacogenetics, pharmacogenomics, treatment response

## Abstract

**Background:**

Chronic obstructive pulmonary disease (COPD) is a heterogeneous respiratory disease in which patients may differ in terms of clinical course and response to drug treatment. Genetic variability may contribute to these differences by influencing drug response and clinical outcomes. This systematic review aimed to summarize the available evidence on the association between genetic variants and clinical outcomes or treatment response in patients with COPD receiving LABA- and/or LAMA-based therapies, as well as pharmacogenetic response to combination inhalants.

**Methods:**

A systematic search was conducted in the PubMed and Scopus databases, as well as in the gray literature, for studies published between 2015 and 2026. Studies were considered eligible if they included adults with COPD receiving LABA and/or LAMA treatment, including combination regimens, and evaluated genetic variants in relation to therapeutic response. Additionally, studies focusing on disease phenotypes rather than specific pharmacogenetic markers were also included. Study selection and data extraction were performed by two independent reviewers. Risk of bias was assessed using the Newcastle-Ottawa Scale (NOS) and Cochrane Risk of Bias 2 (RoB 2) tool.

**Results:**

A total of 51 records were identified, and eight studies were included. Genetic variants in the *ADRB2, FAM13A, SUMF1, MAPK8, AZU1, CSE1L*, and *CCL2* genes were investigated in relation to lung function, disease severity, and treatment response. *ADRB2* variants (rs1042713, rs1042714, and rs1800888) were associated with lung function and exacerbation risk, although results regarding bronchodilator treatment response were inconsistent. Regarding other genetic variants, *SUMF1* rs793391 emerged as a candidate variant in patients treated with combined bronchodilators and inhaled corticosteroids (ICS). However, these findings are based on limited data and currently lack independent replication, warranting cautious interpretation. Furthermore, findings from large GWAS revealed novel candidates associated with treatment outcomes, though these results were not replicated in subsequent independent enome-Wide Association Study analyses. Due to the limited number of studies and considerable heterogeneity, no meta-analysis was performed.

**Conclusion:**

This review highlights the latest advances in COPD pharmacogenetics by synthesizing available evidence on patient responses to bronchodilators and corticosteroids. By mapping key methodological factors, the review provides valuable insight into optimizing future research.

**Systematic Review Registration:**

www.crd.york.ac.uk/prospero/, identifier CRD420261392469.

## 1 Introduction

Chronic obstructive pulmonary disease (COPD) is a major cause of morbidity and mortality globally, incurring substantial healthcare and economic expenses (Chen et al., 2023). The predominant symptoms of COPD are dyspnea, sputum production, and a persistent cough occurring in adults only, usually worsening with time (World Health Organization, 2026). The main pathophysiological characteristics of COPD are air trapping and expiratory airflow restriction, which result in lung hyperinflation (O’Donnell and Laveneziana, 2006). The latter contributes to dyspnea leading to activity limitations during stable disease, as well as worsening symptom deterioration during exacerbations. COPD exacerbations may be triggered by single or combined factors, including respiratory infections (bacterial, viral, or co-infections), environmental pollutants, and unidentified elements. These episodes are associated with increased airway and systemic inflammation, as well as physiological alterations, particularly the onset of hyperinflation (Wedzicha and Seemungal, 2007; Gold, 2026). As the disease progresses, dyspnoea and the frequency and severity of exacerbations escalate, resulting in a decline in patients’ daily activities and health-related quality of life (HRQoL) (Wacker et al., 2016; O’Donnell and Parker, 2006).

The primary therapeutic objectives for stable COPD, according to the Global Initiative for Chronic Obstructive Lung Disease (GOLD), are to alleviate symptoms and diminish the risk of future exacerbations (Gold, 2026). The pharmacological treatment of moderate to severe stable COPD focuses on long-acting bronchodilators, comprising two medication classes: long-acting β_2_ agonists (LABAs) and long-acting muscarinic antagonists (LAMAs) (NICE, 2026; Roche et al., 2013). The simplified ABE assessment scheme guides initial maintenance therapy: long-acting bronchodilator monotherapy (LAMA or LABA) is indicated for patients with low symptom burden and low exacerbation risk (Group A), whereas dual long-acting bronchodilator therapy (LABA + LAMA) is preferred as first-line treatment for patients with high symptom burden (Group B) or a history of exacerbations (Group E, defined by ≥ 1 moderate or severe exacerbation). Importantly, inhaled corticosteroids (ICS), typically as triple therapy (LABA + LAMA + ICS), are used as a strategy specifically targeted at patients with an exacerbation-prone phenotype and elevated blood eosinophil counts (≥300 cells/µL) to mitigate type-2 airway inflammation (Gold, 2026; Miravitlles et al., 2022).

The combination of LABA and LAMA provides a synergic effect with long duration of action (Nici et al., 2020). LABAs act by stimulating β_2_-adrenergic receptors on airway smooth muscles to functionally antagonize bronchoconstriction, whereas LAMAs block the bronchoconstrictor effects of acetylcholine on M_3_ muscarinic receptors (Ejiofor and Turner, 2013). It has been demonstrated that dual therapy using LABA/LAMA provides greater benefits than monotherapy in terms of symptom management and different clinical outcomes such as dyspnoea, HRQoL, exacerbations and a significant improvement in lung function measured by the forced expiratory volume in 1 s (FEV_1_) (Miravitlles et al., 2022; Villar Martínez et al., 2024)**.**

Despite the different treatment options, many COPD patients remain symptomatic or show significant variability in treatment response, highlighting the ongoing need for novel therapies in this population (Hersh, 2010). It is well established that COPD is a heterogeneous condition with various disease subtypes. A pharmacogenomic approach plays a key role as it may identify which COPD patients are most likely to respond to both current and novel COPD treatments (Hersh, 2019). Although specific single nucleotide polymorphisms (SNPs) have been linked to variable bronchodilator responsiveness (Matera et al., 2023), prior evidence synthesis, such as earlier systematic reviews focused predominantly on candidate gene analyses like β_2_-adrenergic receptor gene (*ADRB2)*, showed inconclusive associations and left substantial gaps regarding modern therapeutic strategies.

To bridge these limitations, this systematic review provides an updated and comprehensive synthesis of pharmacogenomic evidence. Beyond isolated candidate gene studies, this review expands the current knowledge base by incorporating Genome-Wide Association Study (GWAS) data, dual LABA/LAMA combination regimens, and corticosteroid-containing inhalants. Furthermore, by integrating grey literature and focusing on modern pharmacogenomic evidence synthesis, this work offers a broader perspective on how both specific genetic markers and clinical or disease phenotypes influence treatment response in COPD management.

## 2 Materials and methods

### 2.1 Search strategy

This systematic review was conducted according to the Preferred Reporting Items for Systematic Reviews and Meta-Analyses (PRISMA 2020) guidance (Page et al., 2021). A preliminary investigation was conducted in 2025 by two group members of the research team to define the study design and develop the search strategy. The final comprehensive search was conducted in PubMed and Scopus for literature published between January 2015 to May 2026 on molecular pharmacogenetics and clinical outcomes related to LAMA and LABA therapies and combination regimens in patients with COPD. PubMed and Scopus were selected as the principal bibliographic databases because they provided broad coverage of the biomedical and multidisciplinary literature and were accessible to the research team. In addition, we also conducted a search in grey literature using the sources Clinicaltrials.gov (https://clinicaltrials.gov), Clinical Trials EU (https://euclinicaltrials.eu/), Open Access Thesis Dissertations (OATD) and TESEO database (Spanish Ministry of Education database for doctoral thesis).

The Patient problem, Intervention, Comparison, and Outcome (PICO) framework was used to develop the literature search strategy. Briefly, this consisted by using the terms (“COPD” OR “chronic obstructive pulmonary disease”) AND (“LAMA” OR “long-acting muscarinic antagonist” OR “LABA” OR “Long-Acting Beta2-Agonists” OR “Long-acting Adrenergic Beta Agonist” OR “long-acting beta agonist” OR “dual bronchodilator”) AND (“pharmacogenetics” OR “pharmacogenomics” OR “polymorphism”) among others. The full list of terms used as queries is detailed in the protocol registered in the PROSPERO database (https://www.crd.york.ac.uk/prospero/; registration number CRD420261392469) and Supplementary Table S1.

### 2.2 Eligibility criteria

Studies were considered eligible if they met the following criteria: (i) studies involving adults diagnosed with COPD, regardless of sex, ethnic origin, geographical location, social context, or associated comorbidities; (ii) treatment with LABA and/or LAMA within the context of a pharmacogenetic or pharmacogenomic approach; (iii) studies assessing germline genetic variants in relation to treatment-related clinical outcomes, baseline COPD phenotype, disease severity, clinical course, or risk of exacerbations; and (iv) studies reporting clinical outcomes related to treatment efficacy, treatment non-response, or COPD clinical course. If data on sex, ethnicity, geographical location, social context or associated comorbidities were collected, checks were carried out to ensure that these were included as covariates in the statistical model.

This systematic review included three areas of evidence: (i) the association between genetic variation and the COPD phenotype, (ii) the association between genetic variation and the risk of COPD exacerbations, and (iii) the pharmacogenetic response to treatment, which was defined as evidence that the effect of a pharmacological treatment on a clinical outcome differed according to genetic variation, as assessed through an interaction between genotype and treatment or an equivalent comparative analysis. Associations between genetic variants and clinical outcomes observed solely among treated patients, without evidence of differential treatment effects according to genotype, were not considered sufficient to establish a pharmacogenetic treatment-response effect.

Regarding the study design, observational studies (cohort-based and case-control studies), randomised and non-randomised clinical trials, and articles obtained from relevant grey literature sources were included.

On the contrary, studies were excluded if they met any of the following criteria: systematic and bibliographic reviews, meta-analyses, book chapters, non-human studies, and articles published in languages other than English or Spanish, and studies where the full text was not available.

### 2.3 Study selection

All records identified through the database searches were downloaded and uploaded to the Rayyan online collaborative systematic review platform (Ouzzani et al., 2016). Duplicate records were identified and removed using the same platform. Two authors independently screened the titles and abstracts of all studies identified through the search strategy to assess their eligibility. The same two reviewers independently assessed the full-text articles to determine whether they met the eligibility criteria. The disagreements between reviewers were resolved through discussions and any remaining discrepancies were resolved by the lead reviewer. Trial registry records were screened using the same eligibility criteria as published reports. Registry records and associated publications, when available, were cross-checked to identify records referring to the same study and avoid double counting. Multiple reports derived from the same study population were linked and treated as separate reports of the same cohort rather than as independent studies.

### 2.4 Data extraction

Data extraction was performed independently by two reviewers using a predefined data extraction form. Discrepancies between reviewers were discussed and resolved by consensus.

The following variables were systematically extracted from each eligible study using a structured data collection form:

- **Study and bibliographic data**: title, publication year, journal, authors, DOI, and keywords.
- **Population and intervention**: target population characteristics (number of participants, ancestry/ethnacy and discovery/replication status) and the specific treatment administered.
- **Genetic methodological data**: gene name, gene function, reference SNP cluster IDs (rsID), genotyping approach and quality control procedures, SNP call rate, Hardy-Weiberg equilibrium (HWE), linkage disequilibrium (LD), minor allele frequency (MAF) or genotype frequencies, genetic model (codominant, dominant, recessive, over-dominant, or per-allele model), allele designation (reference/alternative) and effect allele.
- **Clinical outcomes**: treatment-related outcomes were classified into the following domains: (i) exacerbation response, including changes in the frequency or rate of COPD exacerbations, time to first exacerbation, and exacerbation-related hospitalisations; (ii) lung-function response, including changes in FEV_1_, Forced Vital Capacity (FVC), and FEV_1_/FVC; (iii) patient-reported outcome response, including changes in St. George’s Respiratory Questionnaire (SGRQ), COPD Assessment Test (CAT), and modified Medical Research Council (mMRC) scores and general health status; (iv) treatment non-response, defined as the absence of clinical response to pharmacological treatment when explicitly assessed by the original study; and (v) safety outcomes, including treatment-related adverse events, when reported. Other measures related to the clinical course of COPD, such as antibiotic and systemic glucocorticoid use, were recorded separately. No pharmacogenetic safety outcomes were reported among the studies ultimately included in the review.
- **Statistical analysis:** hazard ratios (HR), odds ratios (OR) and other effect estimates, together with their corresponding statistical significance (*p*-values and/or confidence intervals (95% CI)). Population stratification correction and multiple-testing correction was also extracted.

### 2.5 Data analysis

Given the substantial methodological, clinical, and statistical heterogeneity among the included studies and the lack of comparable effect estimates, a quantitative meta-analysis was not performed (Campbell et al., 2020). Specifically, quantitative pooling was precluded by incomplete SNP reporting across studies due to an insufficient number of independent cohorts per variant, extreme clinical outcome heterogeneity across divergent endpoints, and heterogeneity of statistical metrics derived from non-combinable analytical models. Furthermore, synthesis was limited by severe sample size imbalance, which would cause larger studies to heavily distort statistical weights. Therefore, quantitative synthesis was considered inappropriate, and findings were synthesized narratively.

The results from the various studies were compared to identify consistent findings among genetic variables, treatment groups and clinical outcomes. To provide a structured interpretation of the heterogeneous evidence, findings were classified according to the type of association evaluated: (i) predictive pharmacogenetic evidence, assessing whether genotype modified treatment response; (ii) prognostic genotype associations, in which genotype was associated with clinical outcomes independently of treatment effects; (iii) disease susceptibility evidence, in which genotype was associated with COPD compared with controls; and (iv) mechanistic or biological evidence, including gene expression or functional findings. This framework was used to distinguish pharmacogenetic treatment-response effects from genetic associations related to prognosis, disease susceptibility, or underlying biological mechanisms.

### 2.6 Risk of bias assessment

The methodological quality and risk of bias (RoB) of the included observational studies were assessed using the nine-star Newcastle-Ottawa Scale (NOS) (OHRI, 2022). The quality assessment comprised three components: selection (with a maximum of four stars), comparability (two stars), and evaluation of the outcome or exposure (three stars). The thresholds for NOS interpretation were as follows: a total score of 7–9 was categorised as ‘high quality/low RoB', 4–6 stars as ‘moderate quality/RoB', and 0–3 stars as ‘poor quality/high RoB’ (Sahu, 2026).

Pharmacogenetic analyses derived from randomized controlled trials were evaluated using the Cochrane Risk of Bias 2 (RoB 2) tool. The assessment considered the methodological characteristics of the parent randomized trials, including bias arising from the randomization process, deviations from intended interventions, missing outcome data, measurement of outcomes, and selection of the reported result (Sterne et al., 2019).

Two independent reviewers performed the risk of bias assessment, and any disagreements were resolved by consensus.

## 3 Results

### 3.1 Study identification and selection

The initial search identified 51 records. Following screening and full-text assessment, eight studies were finally included in the systematic review. The study selection process is presented in the PRISMA flow diagram (Figure 1).

**FIGURE 1:**
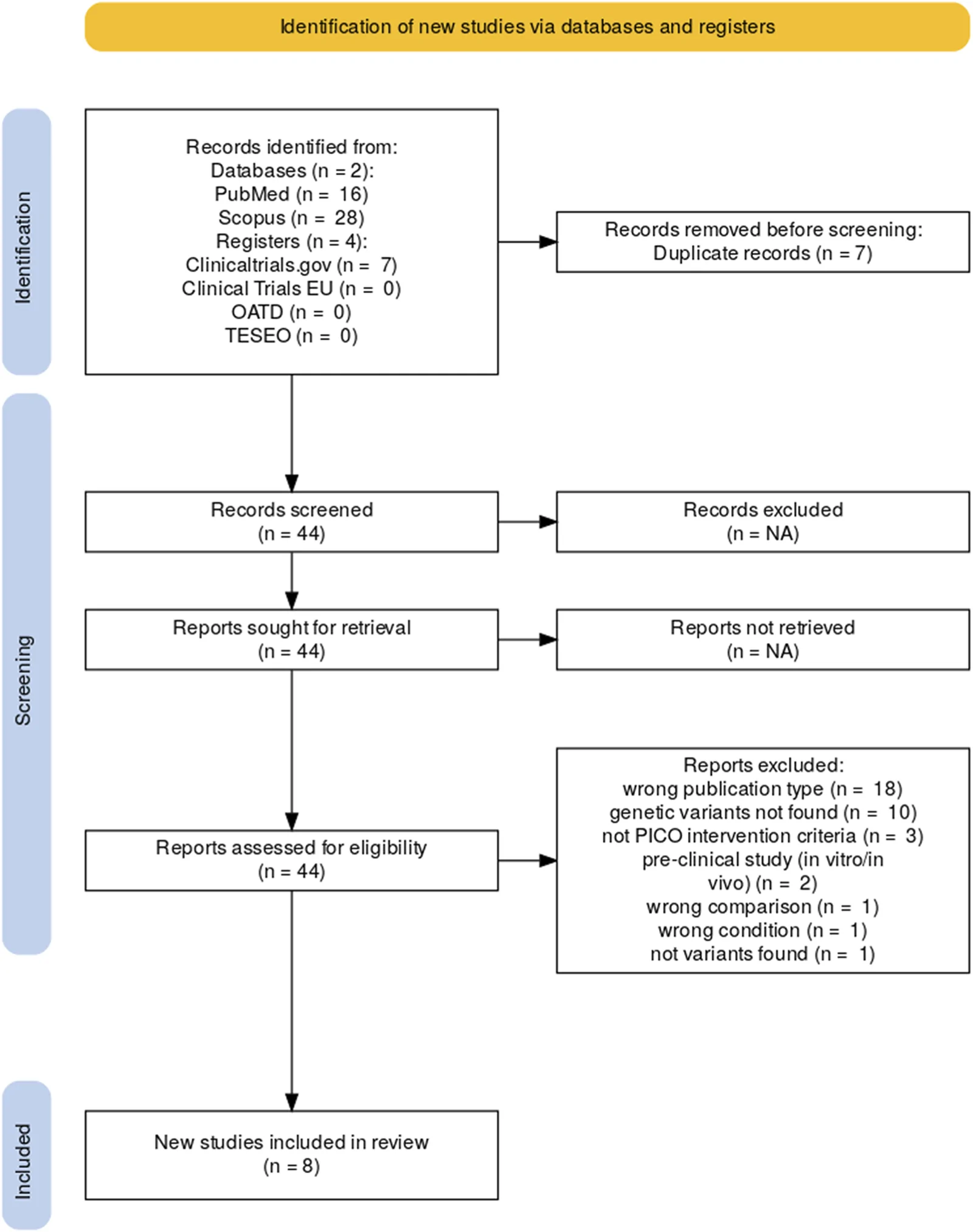
PRISMA flow diagram illustrating the study selection process: fifty-one records identified, seven duplicates removed, forty-four records screened, thirty-eight records excluded, and eight studies included in the final review.

### 3.2 Study characteristics

Eight publications were included in the systematic review, representing seven independent study populations. Four studies publications reported pharmacogenetic analyses using data derived from randomized controlled trials, including pharmacogenetic substudies and *post hoc* pharmacogenetic analyses (Condreay et al., 2016; Ntenti et al., 2025; Hosking et al., 2021; Condreay et al., 2019). Two studies were based on prospective studies (Dmytriiev et al., 2022a; Dmytriiev et al., 2022b), one was an observational cohort study (Karimi et al., 2019), and one was a case-control study (Karimi et al., 2019). Importantly, the two reports by (Dmytriiev et al., 2022a; Dmytriiev et al., 2022b) were derived from the same cohort of 100 patients with COPD treated with tiotropium/olodaterol and evaluated different *ADRB2* variants (Arg16Gly and Gln27Glu); therefore, they were not considered independent cohorts.

The included studies comprised patients with COPD receiving LABA and/or LAMA-based therapies, including monotherapy, dual bronchodilator therapy, and triple therapy with ICS. Sample sizes ranged from 100 to 8,439 patients with COPD, while one case-control study additionally included 150 healthy controls. The studies investigated genetic variants in pharmacologically relevant genes, including *ADRB2,* family with sequence similarity 13 member A *(FAM13A),* sulfatase modifying factor 1 *(SUMF1),* mitogen-activated protein kinase 8 *(MAPK8),* azurocidin 1 *(AZU1),* ADP ribosylation factor guanine nucleotide exchange factor 3 *(ARFGEF3),* chromosome segregation one like *(CSE1L),* staufen double-stranded RNA binding protein 1 *(STAU1)*, and C-C motif chemokine ligand 2 (*CCL2)*. Clinical outcomes included lung function, COPD exacerbations, symptoms and health status, and response to pharmacological treatment. Detailed characteristics of the included studies are presented in Table 1. Likewise genetic methodological characteristics are summarised in Supplementary Table S2. Study populations were recruited in Ukraine (Dmytriiev et al., 2022a; Dmytriiev et al., 2022b), the Netherlands (Karimi et al., 2019), and India (Masood et al., 2026). Population stratification was explicitly addressed using genetic principal components in two studies (Condreay et al., 2016; Hosking et al., 2021), while multiple-testing correction was generally applied in genome-wide and multi-variant analyses. Independent replication was uncommon, with only one study evaluating significant discovery signals in additional clinical trials (Hosking et al., 2021).

**TABLE 1:** Summary of pharmacogenetic and genetic association studies evaluating the relationship between genetic variants and clinical outcomes in COPD.

Design	Population	Treatment	Varians	MAF or genotype frequencies	Clinical outcome	Main findings	Overlap with other included publications	Ref.
Pharmacogenetic analysis of 17 pooled clinical trial data	6075 COPD patients (mean age ∼63 years), mix of current and former smokers. The cohort was multinational; 42% of the PGx sample was recruited in Europe and 28% in the US cohort	LAMA (UMEC), LABA (VI) in monotherapy or combination	*ADRB2* variants Thr164Ile (rs1800888), Arg16Gly (rs1042713), Gln27Glu (rs1042714)	European MAFs were Thr164Ile: 1.2%; Gly16Arg: 37.8%; Gln27Glu: 43.0%	FEV_1_, SGRQ score, and SGRQ responder status	No association of *ADRB2* variants with changes in FEV_1_, FVC, or FEV_1_/FVC in response to treatment. *FAM13A* variants were associated with baseline FEV1/FVC (*p* < 3 × 10^−5^)	No identified overlap	(Condreay et al., 2016)
			*FAM13A* variants: rs7671167, rs2869967, rs1964516, rs1903003	NR				
Prospective study	100 Ukrainian COPD patients stratified according to *ADRB2* Arg16Gly genotype	LAMA/LABA (tiotropium/olodaterol) therapy	*ADRB2* Arg16Gly (rs1042713)	Genotype frequencies: Arg16Arg 23/100 (23%); Arg16Gly 39/100 (39%); Gly16Gly 38/100 (38%)	COPD exacerbations, hospital admissions, mMRC score, CAT score, antibiotic and glucocorticoid use, response to LABA/LAMA therapy	Each Gly allele was associated with a higher risk of exacerbations (OR: 0.25; 95% CI: 0.00–055; *p* = 0.048). After treatment, the number of exacerbations was similar across genotype groups, indicating no differential treatment response	Yes – same cohort as Gln27Glu	(Dmytriiev et al., 2022b)
Observational cohort study	1053 COPD patients (mean age 69.6 ± 9.0 years) from the Rotterdam Study	Inhaled β2-agonist therapy (SABA and LABA)	*ADRB2* variants Arg16Gly (rs1042713) Gln27Glu (rs1042714)	MAF rs1042713 (A): 35% MAF rs1042714 (G): 47%	Risk of COPD exacerbations (moderate/severe) and time to first exacerbation.	Among current β2-agonist users, each Arg allele of rs1042713 was associated with a 30% lower exacerbation risk (HR: 0.70; 95% CI: 0.59–0.84; *p* = 9.2 × 10^−5^), and each Gln allele of rs1042714 with a 20% lower risk (HR:0.80; CI: 0.59–0.85; *p* = 2.1 × 10^−4^). The Arg16/Gln27 haplotype was also protective	No identified overlap	(Karimi et al., 2019)
Prospective study	100 Ukrainian COPD patients stratified according to *ADRB2* Gln27Glu genotype	LAMA/LABA (tiotropium/olodaterol) therapy	*ADRB2* Gln27Glu (rs1042714)	Genotype frequencies Gln27Gln 35/100 (35%); Gln27Glu 39/100 (39%); Glu27Glu 26/100 (26%)	COPD severity (GOLD), exacerbations, hospital admissions, mMRC score, CAT score, antibiotics and glucocorticoids use, response to tiotropium/olodaterol therapy	Carriers of the Glu allele were more frequently classified as GOLD stage D, which corresponds to a more severe disease phenotype (p = 0.035). However, after 1 year of treatment, exacerbations, hospitalizations, and symptom scores improved similarly across all genotype groups, indicating no differential treatment response	Yes – same cohort as Arg16Gly	(Dmytriiev et al., 2022a)
Pharmacogenetic substudy of a randomized controlled trial data	165 Caucasian COPD patients from the HISTORIC study	LAMA/LABA/ICS (aclidinium/formoterol/budesonide) vs. LAMA/LABA/placebo (aclidinium/formoterol/placebo)	*SUMF1* variants: rs793391 T>G rs11915920 C>T	MAF rs793391 (G): 25.1% MAF rs11915920 (C): 45.2%	FEV_1_ change after 12 months of ICS treatment, SGRQ, CAT score, general health status	Among patients receiving LAMA/LABA/ICS, heterozygotes TG for rs793391 showed a significantly greater improvement in FEV_1_ over 12 months compared with those receiving LAMA/LABA/placebo (*p* = 0.002), with a clinically relevant mean reduction of 5.5 points in the SGRQ score	No identified overlap	(Ntenti et al., 2025)
Post hoc pharmacogenetic GWAS of 23 randomized controlled trial data	8,439 patients with moderate to severe COPD (from the CTT116855 trial; 2,201 additional patients for replication); Multiethnic PGx population; 83% White European	ICS/LAMA/LABA (FF/UMEC/VI) vs. ICS/LABA (FF/VI) vs. LAMA/LABA (UMEC/VI)	*MAPK8* variant: rs56195836	MAF rs56195836 (T): 10%	Annual rate of moderate/severe exacerbations, exacerbation rate in patients with baseline eosinophils ≥150 cells/µL, change in trough FEV_1_, change in SGRQ, clinically important deterioration (CID).	*MAPK8* rs56195836 was associated with increased exacerbation rate in patients with eosinophils ≥150 cells/µL (*p* = 1.8 × 10^−8^) receiving FF-containing therapy; *AZU1* rs1962343 was associated with reduced exacerbation rate in frequent exacerbators receiving FF/VI (*p* = 1.14 × 10^−8^)	No identified overlap	(Hosking et al., 2021)
			*AZU1* variant: rs1962343	MAF rs1962343 (T): 24%				
Post hoc pharmacogenetic GWAS of pooled clinical trial data	2005 COPD patients from 10 clinical trials (1,188 evaluated for SGRQ response)	ICS/LABA (fluticasone propionate/salmeterol (FSC))	7 low-frequency variants at a chromosome 20 locus, including *CSE1L* rs2869693	Low MAF variants (MAF ≈1%)	FEV_1_, SGRQ score and SGRQ responder status	7 correlated low-frequency variants were associated with change in SGRQ scores, but the associations did not reach genome-wide significance levels. No genetic association with FEV_1_ response was identified	No identified overlap	(Condreay et al., 2019)
Case-control study	150 North Indian COPD patients and 150 healthy controls. Treatment response subset (n = 46)	ICS/LABA (budesonide/formoterol)	*CCL2* variant: rs3760399 A/G	MAF (G) = 19% (controls); MAF (G) 29% (patients)	Clinical response to budesonide/formoterol treatment (responders vs. non-responders)	rs3760399 GG genotype was associated with COPD susceptibility (OR:3.929; *p* = 0.0033) but not with response to budesonide/formoterol. Higher *CCL2* expression, rather than genotype, was independently associated with treatment non-response (OR: 4.956; *p* = 0.031)	No identified overlap	(Masood et al., 2026)

Abbreviations: Chronic Obstructive Pulmonary Disease (COPD); Pharmacogenomics (PGx); Forced Expiratory Volume in 1 s (FEV1); Forced Vital Capacity (FVC); St. George’s Respiratory Questionnaire (SGRQ); COPD, Assessment Test (CAT); modified Medical Research Council (mMRC); Hazard Ratio (HR); Odds Ratio (OR); Confidence Interval (CI); Short-Acting β_2_-Agonist (SABA); Long-Acting Muscarinic AntagonisT (LAMA); Long-Acting β_2_-Agonist (LABA); Inhaled Corticosteroids (ICS); minor allele frequency (MAF) are presented as percentages for consistency across studies; Global Initiative for Chronic Obstructive Lung Disease (GOLD); Genome-Wide Association Study (GWAS); NR, not reported; Ref., references.

### 3.3 Narrative synthesis of genetic evidence

#### 3.3.1 Predictive pharmacogenetic evidence

In the study by Condreay et al. (2016), none of the three *ADRB2* variants investigated (Gly16Arg [rs1042713], Gln27Glu [rs1042714], and Thr164Ile [rs1800888]) showed a significant association with changes in FEV_1_, FVC, or FEV_1_/FVC in response to umeclidinium, vilanterol, or their combination (Condreay et al., 2016).

In a *post hoc* pharmacogenetic analysis conducted by Condreay et al. (2019), genetic associations with the response to fluticasone propionate/salmeterol (FSC) were evaluated using changes in the St. George Respiratory Questionnaire (SGRQ) score and trough FEV_1_ (Condreay et al., 2019). A locus represented by seven low-frequency variants (allele frequency less than approximately 1%), including rs2869693 in the *CSE1L* region, was significantly associated with change from baseline in the SGRQ score (*p* GWAS ≤5 × 10^−8^). However, genetic associations with binary SGRQ scores and FEV_1_ response did not reach genome-wide significance levels (Ntenti et al., 2025).

In the pharmacogenetic analysis conducted by Ntenti et al. (2025), the rs793391 variant of the *SUMF1* gene was associated with response to LAMA/LABA treatment combined with inhaled corticosteroids (Ntenti et al., 2025). Among patients receiving LAMA/LABA/ICS, heterozygotes for rs793391 (TG) showed a significantly greater improvement in FEV_1_ over 12 months compared with those receiving LAMA/LABA/placebo (*p* = 0.002), with a clinically relevant mean reduction of 5.5 points in the SGRQ score. In contrast, no statistically significant treatment-related improvements were observed for the TT or GG genotypes; although interpretation of the GG subgroup was limited by the very small number of carriers (*n* = 9) (Sterne et al., 2019).

In the pharmacogenetic analysis conducted by Hosking et al. (2021), the minor allele rs56195836 of the *MAPK8* gene was significantly associated with a higher rate of moderate-to-severe exacerbations during treatment in patients receiving fluticasone furoate (FF) who had baseline blood eosinophil counts of ≥150 cells/μL (*p* = 1.81 × 10^−8^) (Hosking et al., 2021). The same direction of effect was observed in the two treatment groups that included FF, FF/UMEC/VI and FF/VI but not in the UMEC/VI group. In a *post hoc* analysis, the *AZU1* minor T allele (rs1962343) was significantly associated with a reduction in the annual rate of moderate-to-severe exacerbations during treatment among patients receiving FF/VI who had a history of frequent moderate exacerbations (*p* = 1.14 × 10^−8^). However, none of these associations were supported in the independent follow-up analyses (Condreay et al., 2016).

#### 3.3.2 Prognosis genotype associations

In the study by Condreay et al. (2016), treated patients carrying *FAM13A* variants (rs7671167, rs2869967, rs1964516, and rs1903003) were significantly associated with baseline FEV_1_/FVC ratio (*p* < 3 × 10^−5^) (Sahu, 2026).

In the experimental prospective study by the Arg16Gly variant of the *ADRB2* gene (rs1042713) was associated with the clinical course of COPD (Dmytriiev et al., 2022b). The presence of copies of the Gly allele was associated with differences in hospital admissions related to exacerbations (OR: 0.25; 95% CI: 0.00–0.55; *p* = 0.048), as well as with increased antibiotic use, both in terms of the number of treatment cycles (between the Arg16Arg and Arg16Gly groups [*p* = 0.018] and between the Arg16Arg and Gly16Gly groups [*p* = 0.015]) and the total duration of treatment (*p* = 0.037). In addition, a weak association was observed between Gly copies and the duration of glucocorticoid treatment (*p* = 0.045). However, the Arg16Gly allele did not significantly alter the clinical response to LAMA/LABA treatment in terms of exacerbations, hospitalisations or improvements in mMRC and CAT scores (Dmytriiev et al., 2022b).

In the Rotterdam Study, evaluated the association between *ADRB2* gene polymorphisms and the risk of COPD exacerbations among users of inhaled β_2_ agonists, including both short-acting (SABA) and long-acting β_2_ agonists (LABA) (Karimi et al., 2019). The probability of experiencing exacerbations was reduced by 30% for each copy of the Arg allele of rs1042713 (HR: 0.70; 95% CI: 0.59–0.84; *p* = 9.2 × 10^−5^) and by 20% for each copy of the Gln allele of rs1042714 (HR: 0.80; 95% CI: 0.69–0.94; *p* = 7.2 × 10^−3^). In addition, carriers of the Arg16/Gln27 haplotype had a lower risk of exacerbations compared with carriers of the Gly16/Glu27 haplotype (HR: 0.70; 95% CI: 0.59–0.85; *p* = 2.1 × 10^−4^). These associations were observed amongst current users of β2-agonists, while no significant associations were identified in the general population, among non-users or among former users. In a sensitivity analysis restricted to LABA-only users, rs1042713 showed a similar association with a reduced risk of exacerbations (adjusted HR: 0.70; 95% CI: 0.48–0.98; *p* = 0.040), whereas no significant association was observed for rs1042714 (adjusted HR: 0.92; 95% CI: 0.67–1.27; *p* = 0.631). However, the study did not formally demonstrate an interaction between genetic variation and response to treatment (Karimi et al., 2019).

In the experimental prospective study by the Gln27Glu variant of the *ADRB2* gene (rs1042714) was associated with COPD severity (*p* = 0.004) (Dmytriiev et al., 2022a). Carriers of the Glu allele were more frequently classified as GOLD stage D, which corresponds to a more severe disease phenotype (*p* = 0.035). These patients also required a significantly higher number of antibiotic courses (*p* = 0.034) and glucocorticoid treatments (*p* = 0.023) at the end of the study. However, treatment with LAMA/LABA was associated with a reduction in symptoms and exacerbations across all genotype categories (*p* < 0.001), with no evidence that the Gln27Glu genotype modified the clinical response to treatment (Dmytriiev et al., 2022a).

#### 3.3.3 Disease susceptibility evidence and mechanistic/biological evidence

In the case-control study by Masood et al. (2026), the *CCL2* rs3760399 GG genotype was significantly more frequent among patients with COPD than healthy controls (13.3% vs. 4.0%; *p* = 0.0033; OR: 3.929, 95% CI 1.58–9.85) (Masood et al., 2026). Similarly, the G allele was more frequent among patients with COPD (29% vs. 19%; *p* = 0.0072; OR: 1.713, 95% CI 1.18–2.53), indicating an association between rs3760399 and COPD susceptibility rather than treatment response. Additionally, *CCL2* expression was also significantly higher in patients with COPD than in controls (*p* < 0.0001), with GG carriers showing higher expression than AA (*p* = 0.0009) and AG (*p* = 0.0368) carriers. Treatment response to budesonide/formoterol was assessed in a subset of 46 patients with COPD (16 responders and 30 non-responders), with no significant differences in rs3760399 genotype or allele frequency between the two groups. In addition, higher *CCL2* expression, rather than the rs3760399 genotype itself, was associated with a lack of response to ICS/LABA treatment (OR: 4.956; *p* = 0.031) (Masood et al., 2026).

### 3.4 Methodological quality and risk of bias

The methodological quality and RoB of the observational included studies were assessed using the nine-star NOS (Figure 2A; Supplementary Tables S3,S4). Among the four studies, only one received eight stars (Karimi et al., 2019), reflecting overall high methodological quality and low risk of bias. The remaining studies obtained lower NOS scores and were therefore considered to have moderate methodological quality and RoB (Dmytriiev et al., 2022a; Dmytriiev et al., 2022b; Masood et al., 2026).

**FIGURE 2:**
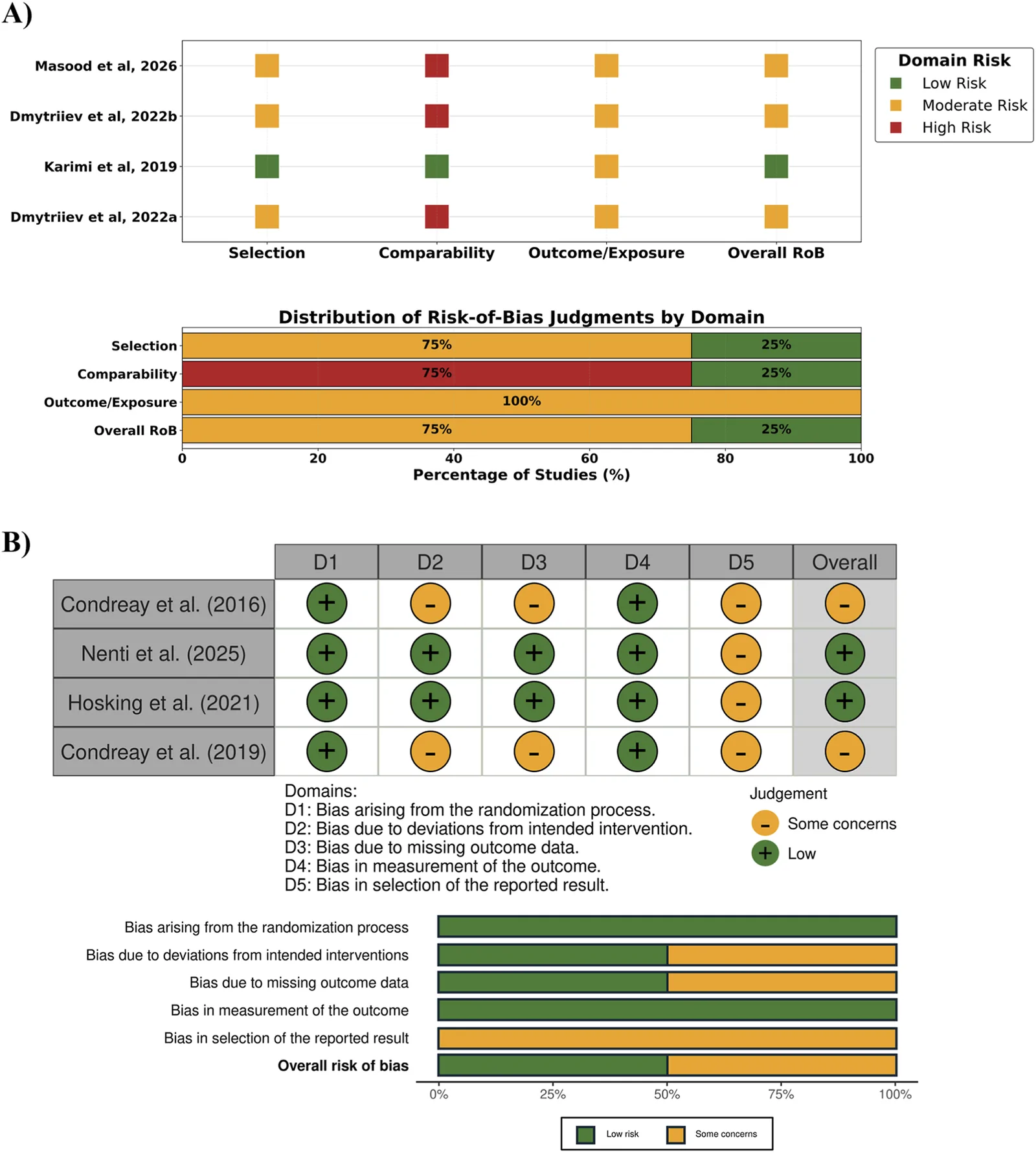
Risk of bias assessment for **(A)** observational studies using NOS and **(B)** pharmacogenetic substudies derived from randomized controlled trials using RoB two.

Regarding pharmacogenetic substudies derived from randomized controlled trials (Figure 2B; Supplementary Table S5), an overall low risk of bias was observed in domains related to randomization, intervention assignment, and outcome measurement, whereas some concerns were identified mainly regarding selection of reported results due to the exploratory nature of pharmacogenetic analyses and the evaluation of multiple genetic variants and clinical outcomes (Condreay et al., 2016; Ntenti et al., 2025; Hosking et al., 2021; Condreay et al., 2019).

## 4 Discussion

Pharmacotherapy with long-acting bronchodilators (LABAs and LAMAs) forms the cornerstone of stable COPD management; however, marked interindividual variability in treatment response persists, underscoring the urgent need for personalized therapeutic strategies. In this systematic review, we comprehensively synthesized current evidence regarding the impact of genetic variations on treatment outcomes and clinical responses to long-acting bronchodilators and corticosteroids in patients with COPD. In the first place, we identified four studies evaluating the influence of *ADRB2* variants in relation to bronchodilator exposure, treatment outcomes, or COPD clinical course, which have yielded inconsistent results (Condreay et al., 2016; Dmytriiev et al., 2022a; Dmytriiev et al., 2022b; Karimi et al., 2019). The gene *ADRB2* codes for the β_2_-adrenergic receptor, a G protein-coupled receptor highly expressed in airway smooth muscle and a major pharmacological target of β_2_-agonists used for bronchodilation in COPD (Rana et al., 2025). It is well-known that *ADRB2* polymorphisms have demonstrated functional properties (Fawcett et al., 2025). Particularly, three coding region polymorphisms, Thr164Ile variant (rs1800888), Arg16Gly (rs1042713), and Gln27Glu (rs1042714) have been shown to affect receptor intracellular signaling after ligand stimulation (Fawcett et al., 2025).

Among the studies evaluating *ADRB2* polymorphisms, the prospective, population-based Rotterdam Study by reported a significant association of *ADRB2* variants (rs1042713 and rs1042714) and the risk of exacerbations among current users of inhaledβ2-agonists, includingboth SABA and LABA exposure (Karimi et al., 2019). A sensitivity analysis restricted to LABA-only users showed a similar association for rs1042713, although no significant association was observed for rs1042714. However, given the observational design of the study, these findings should be interpreted with caution (Karimi et al., 2019). Although the analyses were adjusted for age, sex, smoking status, concomitant ICS use, and daily β2-agonist dose, the observational design does not exclude residual confounding, particularly in relation to disease severity, treatment indication, adherence, and differences in treatment intensity. Furthermore, the study did not formally demonstrate an interaction between genetic variation and treatment.

In a large pooled pharmacogenetic analysis of 17 randomized clinical trials compromising 6,075 patients, Condreay et al. (2016) evidenced that none of the three functional variants examined (Arg16Gly rs1042713, Gln27Glu rs1042714 and Thr164Ile rs1800888) was associated with the clinical response to bronchodilator-treatment, both in monotherapy and in combination (Condreay et al., 2016). In another large population-based phenome-wide analysis using UK Biobank, Fawcett et al. (2025) revealed that the minor T allele of Thr164Ile polymorphism was more related to reduced lung function, but not with susceptibility to COPD. Although the analysis of LABA response was conducted in 3,251 adults with asthma rather than COPD, *ADRB2* coding variants should be considered supportive predictors of LABA response rather than direct evidence regarding pharmacogenetic response in COPD (Fawcett et al., 2025).

Two additional studies by based on the same cohort of 100 Ukrainian patients with COPD, evaluated the influence of the Gln27Glu (rs1042714) and Arg16Gly (rs1042713) polymorphisms on clinical outcomes during 52 weeks of treatment with tiotropium/olodaterol (Dmytriiev et al., 2022a; Dmytriiev et al., 2022b). Although both variants were associated with differences in the clinical course of COPD, these associations did not translate into a differential response to LABA/LAMA therapy. Moreover, after 1 year of tiotropium/olodaterol treatment, exacerbations, hospitalizations, and symptom scores improved similarly across all genotype groups (Dmytriiev et al., 2022a; Dmytriiev et al., 2022b). These findings therefore suggest that *ADRB2* polymorphisms may influence COPD phenotype and disease burden without substantially modifying the clinical efficacy of LABA/LAMA treatment.

On the other hand, the remaining four studiesevaluated additional variants in candidate genes in relation to ICS-containing combination therapies, but likewise failed to identify robust pharmacogenetic predictors. In a *post hoc* exploratory GWAS integrating 10 clinical trials, Condreay et al. (2019) evaluated 2,005 COPD patients treated with combined ICS/LABA treatment (FSC), of whom 1,188 had available quality-of-life data. Although seven correlated low-frequency variants (MAF ∼1%) in a chromosome 20 region encompassing *ARFGEF3, CSE1L*, and *STAU1* were significantly associated with changes in baseline SGRQ score, no genome-wide association was observed for the binary SGRQ response or FEV_1_ improvement (Condreay et al., 2019). Similarly, in a large genetic study integrating data from 23 randomized, double-blind, parallel-group clinical trials and two disease cohorts (n = 19,841), Hosking et al. (2021) further performed one of the largest pharmacogenetic GWAS analyses to date in 8,439 patients with moderate-to-severe COPD from the IMPACT trial (Hosking et al., 2021). Here, two common variants, *MAPK8* rs56195836 and *AZU1* rs1962343, located in genes involved in JNK-mediated stress/apoptotic signaling and inflammation, respectively, showed genome-wide significant associations with triple therapy-related exacerbation outcomes. However, these findings represented initial discovery signals, and neither association was confirmed in independent follow-up analyses. This indicates that these associations should not be considered confirmed pharmacogenetic predictors of treatment response, in line with the authors conclusion that common genetic variants are unlikely to play a major role in predicting AECOPD treatment response (Hosking et al., 2021).

More recently, Masood et al. (2026) conducted a case-control study in a North Indian population comprising 150 COPD patients and 150 healthy controls (Masood et al., 2026). Besides *CCL2* rs3760399 variant was linked to COPD susceptibility, it was not associated with response to budesonide/formoterol therapy in the subset of 46 patients evaluated for treatment response. In contrast, the higher *CCL2* expression was observed among non-responders and was independently associated with treatment non-response, suggesting that *CCL2* expression, rather than rs3760399 genotype, may have potential as a biomarker of response to budesonide/formoterol therapy (Masood et al., 2026). COPD progression is strongly marked by chronic inflammation, in which chemokines play a fundamental role in immune cell recruitment (Xu et al., 2024). Here, *CCL2* (*MCP-1*) emerges as a key regulator within this process. Contrary, Ntenti et al. (2025) provided more direct evidence of a genotype-dependent corticosteroid response (Ntenti et al., 2025). In a pharmacogenetic substudy of the randomized, double-blind, placebo-controlled HISTORIC trial, 165 Caucasian patients with COPD were genotyped for two common SNPs in *SUMF1*, rs11915920 and rs793391. It was observed that carriers of rs793391 variant receiving LAMA/LABA/ICS treatment showed a significantly greater improvement in FEV_1_ change and SGRQ score than carriers receiving LAMA/LABA/placebo (Ntenti et al., 2025). Taken together, these findings suggest a genotype-dependent response to ICS-containing therapy, although the authors considered the results exploratory and emphasised the need for validation in larger, independent cohorts.

*SUMF1* genes encode for sulfatases, which are enzymes that modify proteoglycan chains by removing sulfate groups, being critical for the extracellular matrix (ECM). Impaired expression of *SUMF1* contributes to an overabundance of sulfated glycosaminoglycans (GAGs) deposited in the ECM, leading to lung dysfunction (Ntenti et al., 2024). On this basis, it has been hypothesized that genetic variation in *SUMF1* could influence lung function or COPD-related phenotypes; however, the molecular mechanisms by which these variants might modify response to ICS-containing therapies remain unclear and have not been demonstrated.

Despite some relevant pharmacogenetic associations, the current evidence is insufficient to support routine pharmacogenetic testing to guide LABA-, LAMA-, or ICS-containing therapy in COPD. Furthermore, most report associations have not been independently replicated, and even genome-wide significant signals identified in large pharmacogenetic analyses were not confirmed in independent follow-up cohorts. The included studies also involved different ethnic groups, which restricts how widely the individual findings can be generalised. Before clinical implementation, candidate genetic determinants should therefore be replicated in independent, diverse populations and prospectively validated in studies specifically designed to assess their clinical relevance (Pirmohamed, 2023). Importantly, the predictive application of pharmacogenetics requires demonstration of a treatment-by-genotype interaction rather than an association observed within a single treatment group (Zhai et al., 2026). Prognostic variants may still be clinically informative for risk stratification and disease monitoring, but, without evidence that treatment response differs according to genotype, they should not be interpreted as pharmacogenetic predictors, and used for therapeutic decisions (Zhai et al., 2026).

## 5 Conclusions

This review evaluates eight studies investigating the potential of pharmacogenetic approaches for personalized treatment strategies in symptomatic COPD. However, the current evidence remains insufficient to support routine pharmacogenomic testing to guide LABA-, LAMA-, or ICS-containing therapy. These findings underscore the need for larger, well-designed and independently replicated studies to validate reliable genetic markers and establish their clinical utility in COPD.

## 6 Limitations and strengths

This review has some limitations. First, the small number of eligible studies and the substantial heterogeneity in study designs, populations, genetic variants, pharmacological treatments, and clinical outcomes precluded quantitative synthesis. Second, several included studies had relatively small sample sizes, particularly candidate-gene studies and genotype-defined subgroup analyses, which may have limited the statistical power. In addition, some pharmacogenetic analyses were exploratory or *post hoc*, and independent replication of significant findings was limited. Finally, the eligibility criteria, including the restriction to English- and Spanish-language publications and open-access articles, may have limited the identification of relevant evidence and introduced selection bias.

It is worth highlighting some strengths. First, it addresses a clinically relevant and timely question of precision medicine regarding the genetic determinants of interindividual variability in COPD pharmacotherapy. This review provides an up-to-date synthesis of the available evidence, including analysis of large pharmacogenetic datasets. The review was conducted following a systematic and transparent methodology, in accordance with PRISMA 2020 guidelines and with prospective registration in PROSPERO. Study selection and data extraction were performed using predefined procedures and supported by Rayyan, with independent verification to enhance methodological rigor and reproducibility. In addition to conventional bibliographic databases, the search strategy included multiple sources of gray literature, thereby broadening evidence retrieval and potentially reducing selective retrieval of published studies. Methodological quality and risk of bias were assessed using study design-specific tools, with RoB 2 applied to pharmacogenetic substudies derived from randomized controlled trials and the NOS applied to observational studies, allowing a more appropriate appraisal of the available evidence. Finally, the synthesis adopted a cautious interpretation of findings, explicitly considering inconsistencies across studies, lack of replication of several genetic associations, and methodological and clinical heterogeneity rather than interpreting individual associations as established pharmacogenetic biomarkers.

## Data Availability

The original contributions presented in the study are included in the article/Supplementary Material, further inquiries can be directed to the corresponding author.
