# Supplementary material for: Influence of genetic variants on the response to long-acting β2-agonists (LABAs) and long-acting muscarinic antagonists (LAMAs) and combination therapies in patients with chronic obstructive pulmonary disease (COPD): a systematic review
Source: Front Pharmacol. 2026 Oct 29;17:1973557. doi: 10.3389/fphar.2026.1973557 (PMC13755698; doi:10.3389/fphar.2026.1973557)
Supplement: Supplementary file 1 [file Table1.docx]

**Table S1. Search strategy for each database and gray-literature sources**

| **Database** | **Search strategy** |
| --- | --- |
| **Pubmed** | (("copd"[All Fields] OR "chronic obstructive pulmonary disease"[All Fields] OR "pulmonary disease chronic obstructive"[All Fields]) AND ("laba"[All Fields] OR "Long-Acting Beta2-Agonists"[All Fields] OR "Long-acting Beta Agonist"[All Fields] OR "lama"[All Fields] OR "Long-Acting Muscarinic Antagonists"[All Fields] OR "dual bronchodilator"[All Fields] OR "bronchodilator combination"[All Fields] OR "tiotropium"[All Fields] OR "umeclidinium"[All Fields] OR "glycopyrronium"[All Fields] OR "aclidinium"[All Fields] OR "salmeterol"[All Fields] OR "formoterol"[All Fields] OR "indacaterol"[All Fields] OR "olodaterol"[All Fields] OR "vilanterol"[All Fields]) AND ("pharmacogenetic"[All Fields] OR "pharmacogenomic"[All Fields] OR "pharmacogenetics"[All Fields] OR "pharmacogenomics"[All Fields] OR "polymorphism"[All Fields] OR "variant"[All Fields] OR "mutation"[All Fields] OR "SNP"[All Fields] OR "single nucleotide polymorphism"[All Fields] OR "GWAS"[All Fields] OR "genome-wide association study"[All Fields] OR "WES"[All Fields] OR "whole exome sequencing"[All Fields] OR "whole genome sequencing"[All Fields] OR "WGS"[All Fields] OR "NGS"[All Fields])) AND ((ffrft[Filter]) AND (english[Filter] OR spanish[Filter]) AND (2015:2026[pdat])) |
| **Scopus** | TITLE-ABS-KEY ( ( "copd" OR "chronic obstructive pulmonary disease" OR "pulmonary disease, chronic obstructive" ) AND ( "laba" OR "Long-Acting Beta2-Agonists" OR "Long-acting Beta Agonist" OR "lama" OR "Long-Acting Muscarinic Antagonists" OR "dual bronchodilator" OR "bronchodilator combination" OR "tiotropium" OR "umeclidinium" OR "glycopyrronium" OR "aclidinium" OR "salmeterol" OR "formoterol" OR "indacaterol" OR "olodaterol" OR "vilanterol" ) AND ( "pharmacogenetic" OR "pharmacogenomic" OR "pharmacogenetics" OR "pharmacogenomics" OR "polymorphism" OR "variant" OR "mutation" OR "SNP" OR "single nucleotide polymorphism" OR "GWAS" OR "genome-wide association study" OR "WES" OR "whole exome sequencing" OR "whole genome sequencing" OR "WGS" OR "NGS" ) ) AND ( LIMIT-TO ( DOCTYPE , "ar" ) ) AND ( LIMIT-TO ( LANGUAGE , "English" ) ) AND ( LIMIT-TO ( OA , "all" ) ) AND PUBYEAR > 2014 AND PUBYEAR < 2027 |
| [**Clinicaltrials.gov**](http://clinicaltrials.gov) | - Condition/disease: ("copd" OR "chronic obstructive pulmonary disease" OR "pulmonary disease, chronic obstructive") - Other terms: ("pharmacogenetic" OR "pharmacogenomic" OR "pharmacogenetics" OR "pharmacogenomics" OR "polymorphism" OR "variant" OR "mutation" OR "SNP" OR "single nucleotide polymorphism" OR "GWAS" OR "genome-wide association study" OR "WES" OR "whole exome sequencing" OR "whole genome sequencing" OR "WGS" OR "NGS") - Intervention: ("laba" OR "Long-Acting Beta2-Agonists" OR "Long-acting Beta Agonist" OR "lama" OR "Long-Acting Muscarinic Antagonists" OR "dual bronchodilator" OR "bronchodilator combination" OR "tiotropium" OR "umeclidinium" OR "glycopyrronium" OR "aclidinium" OR "salmeterol" OR "formoterol" OR "indacaterol" OR "olodaterol" OR "vilanterol"). - Filters: all studies; studies with results; study start from 01/01/2015. |
| **Clinical Trials Register EU** | ("copd" OR "chronic obstructive pulmonary disease" OR "pulmonary disease, chronic obstructive") AND ("laba" OR "Long-Acting Beta2-Agonists" OR "Long-acting Beta Agonist" OR "lama" OR "Long-Acting Muscarinic Antagonists" OR "dual bronchodilator" OR "bronchodilator combination" OR "tiotropium" OR "umeclidinium" OR "glycopyrronium" OR "aclidinium" OR "salmeterol" OR "formoterol" OR "indacaterol" OR "olodaterol" OR "vilanterol") AND ("pharmacogenetic" OR "pharmacogenomic" OR "pharmacogenetics" OR "pharmacogenomics" OR "polymorphism" OR "variant" OR "mutation" OR "SNP" OR "single nucleotide polymorphism" OR "GWAS" OR "genome-wide association study" OR "WES" OR "whole exome sequencing" OR "whole genome sequencing" OR "WGS" OR "NGS"). Filters: data range (2015-01-01 to 2026-05-20); Trials with results. |
| **OATD** | ("copd" OR "chronic obstructive pulmonary disease" OR "pulmonary disease, chronic obstructive") AND ("laba" OR "Long-Acting Beta2-Agonists" OR "Long-acting Beta Agonist" OR "lama" OR "Long-Acting Muscarinic Antagonists" OR "dual bronchodilator" OR "bronchodilator combination" OR "tiotropium" OR "umeclidinium" OR "glycopyrronium" OR "aclidinium" OR "salmeterol" OR "formoterol" OR "indacaterol" OR "olodaterol" OR "vilanterol") AND ("pharmacogenetic" OR "pharmacogenomic" OR "pharmacogenetics" OR "pharmacogenomics" OR "polymorphism" OR "variant" OR "mutation" OR "SNP" OR "single nucleotide polymorphism" OR "GWAS" OR "genome-wide association study" OR "WES" OR "whole exome sequencing" OR "whole genome sequencing" OR "WGS" OR "NGS"). Filters: Written in Spanish and English; Earliest date 2015; Latest date 2026. |
| **TESEO** | ("copd" OR "chronic obstructive pulmonary disease" OR "pulmonary disease, chronic obstructive") AND ("laba" OR "Long-Acting Beta2-Agonists" OR "Long-acting Beta Agonist" OR "lama" OR "Long-Acting Muscarinic Antagonists" OR "dual bronchodilator" OR "bronchodilator combination") AND ("pharmacogenetics" OR "polymorphism" OR "variant" OR "mutation" OR "SNP" OR "single nucleotide polymorphism"). Filters: from 2015 to 2026. |

**Table S2. Genetic methodological characteristics of the included studies**

| **Study** | **Ancestry** | **Genotype / allele frequencies** | **HWE and LD** | **Genotyping approach and genetic QC** | **SNP call rate** | **Genetic model** | **Reference/**  **alternative allele designation** | **Population stratification correction** | **Multiple-**  **testing correction** | **Discovery / replication status** |
| --- | --- | --- | --- | --- | --- | --- | --- | --- | --- | --- |
| Condreay et al. (2016) [(1)](https://sciwheel.com/work/citation?ids=18892976&pre=&suf=&sa=0) | Exact ancestry/ethnicity distribution was not reported. The cohort was multinational; 42% of the PGx sample was recruited in Europe and 28% in the US cohort. | European MAFs were:  Thr164Ile: 1.2%; Gly16Arg: 37.8%; Gln27Glu: 43.0% | NR | Axiom® BioBank genotyping arrays. QC procedures were not described. | SNP call rate NR. Overall PGx consent rate was 87.5% | Per-allele model | NR | Yes; adjustment for the first 10 genetic principal components (PC1–PC10). | Yes | No independent replication |
| Dmytriiev et al. (2022) [(2)](https://sciwheel.com/work/citation?ids=18886971&pre=&suf=&sa=0) | Participants were recruited at a single centre in Vinnytsia, Ukraine. | Genotype frequencies: Arg16Arg 23/100 (23%); Arg16Gly 39/100 (39%); Gly16Gly 38/100 (38%). | NR | Specific genotyping assay and technical QC procedures were not described. | NR | Genotype comparisons plus per-allele contribution | NR | NR | No | No independent replication |
| Karimi et al. (2019) [(3)](https://sciwheel.com/work/citation?ids=19023379&pre=&suf=&sa=0) | Participants belonged to the population-based Rotterdam Study | MAF rs1042713 (A): 35%  MAF rs1042714 (G): 47% | Both SNPs in HWE and in LD with r2 = 0.47 (D’= 1). | Illumina 500(+duo) and Human 610-Quad BeadChips; imputation using 1000 Genomes phase 1 v3/MACH; imputation quality >0.99 for both SNPs. | NR | Additive model; haplotype analysis also performed. | NR | NR | Yes. Bonferroni | No independent replication |
| Dmytriiev et al. (2022) [(4)](https://sciwheel.com/work/citation?ids=19033674&pre=&suf=&sa=0) | Participants were recruited at a single centre in Vinnytsia, Ukraine. | Genotype frequencies:  Gln27Gln 35/100 (35%); Gln27Glu 39/100 (39%); Glu27Glu 26/100 (26%). | NR | Real-time PCR. QC procedures were not described. | NR | Genotype comparisons plus per-allele contribution | NR | NR | No | No independent replication |
| Ntenti et al. (2025) [(5)](https://sciwheel.com/work/citation?ids=18886967&pre=&suf=&sa=0) | Caucasian patients | MAF rs793391 (G): 25.1%  MAF rs11915920 (C): 45.2% | Both SNPs in HWE. They were not in strong LD (r2=0.145, D’=-0.39) | Real-time PCR with Taqman probes. QC procedures were not described. | NR | Dominant, recessive and over-dominant models. | rs793391 (T reference; G alternative). rs11915920 (NR) | NR | Yes. Dunnett–Hsu method | No independent replication |
| Hosking et al. (2021) [(6)](https://sciwheel.com/work/citation?ids=18886972&pre=&suf=&sa=0) | Multiethnic PGx population; 83% White European. | MAF rs56195836 (T): 10%  MAF rs1962343 (T): 24% | NR | 10 million genome-wide variants plus 23 candidate variants; only subjects passing genotyping QC included. | NR | Per-allele contribution | NR | Yes; 10 genetic principal components included in all models. | Yes; corrected for the number of genetic variants (PGx GWAS: p≤2.5×10⁻⁸; candidate variants: p≤0.001), but not for multiple endpoints, treatments, or subgroup analyses. | Discovery analysis with independent follow-up. Significant MAPK8 and AZU1 associations were not reached. |
| Condreay et al. (2019) [(7)](https://sciwheel.com/work/citation?ids=14730691&pre=&suf=&sa=0) | Exact ancestry/ethnicity distribution was not reported; the PGx population was pooled from 10 clinical trials. | Significant chromosome 20 locus comprised seven correlated variants with MAF ≈1%. Complete genotype frequencies NR. | NR | GWAS. rs2869693 directly genotyped; six associated variants imputed. QC procedures were not described. | NR | Additive | NR | NR | Yes. Genome-wide significance threshold p≤5×10⁻⁸. | Post hoc exploratory discovery analysis; no independent replication. |
| Masood et al. (2026) [(8)](https://sciwheel.com/work/citation?ids=19066615&pre=&suf=&sa=0) | North Indian cohort | MAF rs3760399 (G)= 19% (controls); MAF rs3760399 (g) 29% (patients) | Yes, in controls | PCR-RFLP. QC procedures were not described. | NR | Codominant and per-allele model | AA = statistical reference genotype; A = statistical reference allele in the reported association analyses. | NR | No | No independent replication |

HWE, Hardy–Weinberg equilibrium; LD, linkage disequilibrium; MAF, minor allele frequency; NR, not reported; PC, principal component; PGx, pharmacogenetics; QC, quality control; RFLP, restriction fragment length polymorphism; SNP, single-nucleotide polymorphism.

**Table S3. Newcastle-Ottawa Scale (NOS) tool for observational case-control studies.**

|  | **SELECTION**  **(Max 4 stars)** | | | | **COMPARABILITY (Max 2 stars)** | **EXPOSURE**  **(Max 3 stars)** | | |  |
| --- | --- | --- | --- | --- | --- | --- | --- | --- | --- |
| Reference (Authors, year) | Case Definition  (0 to 1 star) | Cases Representativeness  (0 to 1 star) | Controls Selection  (0 to 1 star) | Controls Definition  (0 to 1 star) | Confounding Factors (0-2 stars) | Exposure Ascertainment  (0 to 1 star) | Same Ascertainment Method (0 to 1 star) | Non-Response Rate  (0 to 1 star) | **Total Quality Scores**  **(Max 9 stars)** |
| Masood et al. (2026) [(8)](https://sciwheel.com/work/citation?ids=19066615&pre=&suf=&sa=0) | * |  |  | * |  | * | * |  | 4 (moderate quality/RoB) |

7-9 stars → high quality → low risk of bias (RoB); 4-6 → moderate quality → moderate RoB; 0-3 → poor quality → high RoB

**Table S4. Newcastle-Ottawa Scale (NOS) tool for observational cohort studies.**

|  | **SELECTION**  **(Max 4 stars)** | | | | **COMPARABILITY (Max 2 stars)** | **OUTCOME**  **(Max 3 stars)** | | |  |
| --- | --- | --- | --- | --- | --- | --- | --- | --- | --- |
| Reference (Authors, year) | Representativeness  (0 to 1 star) | Non-Exposed Cohort Selection  (0 to 1 star) | Exposure Ascertainment  (0 to 1 star) | Outcome at Study Start  (0 to 1 star) | Confounding Factors (0-2 stars) | Outcome Assessment  (0 to 1 star) | Follow-Up Period  (0 to 1 star) | Follow-Up Adequacy  (0 to 1 star) | **Total Quality Scores**  **(Max 9 stars)** |
| [Dmytriiev](https://sciwheel.com/work/bibliography/18886971) et al. (2022) [(2)](https://sciwheel.com/work/citation?ids=18886971&pre=&suf=&sa=0) |  | * | * |  |  | * | * |  | 4  (moderate quality/RoB) |
| Karimi et al. (2019) [(3)](https://sciwheel.com/work/citation?ids=19023379&pre=&suf=&sa=0) | * | * | * | * | ** | * | * |  | 8  (high quality/low RoB) |
| Dmytriiev et al. (2022) [(4)](https://sciwheel.com/work/citation?ids=19033674&pre=&suf=&sa=0) |  | * | * |  |  | * | * |  | 4  (moderate quality/RoB) |

7-9 stars → high quality → low risk of bias (RoB); 4-6 → moderate quality → moderate RoB; 0-3 → poor quality → high RoB

**Table S5. Cochrane risk-of-bias tool for randomized trials (RoB 2)**

| **RoB 2: Condreay et al. (2016)** [**(1)**](https://sciwheel.com/work/citation?ids=18892976&pre=&suf=&sa=0) | | |
| --- | --- | --- |
| **Bias** | **Authors’ judgement** | **Risk of bias judgement** |
| Domain 1: Risk of bias arising from the randomization process | Low risk of bias | The participants included in the analysis originated from randomized clinical trials, preserving the benefits of random allocation. No evidence of baseline imbalance or problems with the randomization process was identified. Although detailed information regarding allocation concealment from all contributing trials was not available, there was no indication of bias arising from the randomization procedure. |
| Domain 2: Risk of bias due to deviations from the intended interventions (*effect of assignment to intervention*) | Some concerns | The randomized interventions were not modified by the pharmacogenetic analysis, and no relevant deviations from assigned treatments were identified. However, the genetic analyses were conducted in a subset of randomized participants with available DNA samples and successful genotyping, which may introduce selection related to genetic sample availability. Therefore, some concerns remain regarding whether the analysed population fully represents the original randomized population. |
| Domain 3: Missing outcome data | Some concerns | Missing genetic data represented a potential limitation because only participants with available samples and successful genotyping were included in the pharmacogenetic analyses. Although the authors reported comparability between the genetic analysis population and the original intent-to-treat population, selection bias due to availability of genetic material cannot be completely excluded. |
| Domain 4: Risk of bias in measurement of the outcome | Low risk of bias | The outcomes evaluated were clinically established measures collected within randomized clinical trials. Outcome assessment was unlikely to be influenced by knowledge of treatment allocation, and no evidence of differential measurement between groups was identified. |
| Domain 5: Risk of bias in selection of the reported result | Some concerns | The main concern relates to the exploratory nature of the pharmacogenetic analysis. Multiple clinical outcomes and large numbers of genetic variants were evaluated, increasing the possibility of selective reporting or analytical flexibility. Although appropriate genome-wide significance thresholds were applied, the absence of a clearly prespecified analysis plan limits certainty regarding selective reporting. |
| Other bias | None to report | None to report |
| Overall risk of bias | Some concerns | The study benefits from being derived from randomized controlled trials, with low risk regarding treatment allocation and outcome measurement. However, some concerns remain due to the secondary nature of the pharmacogenetic analysis, including restriction to participants with available genetic samples, potential selection bias related to genetic data availability, and multiple genetic and clinical analyses performed. These limitations are inherent to many exploratory pharmacogenetic studies and should be considered when interpreting the reported associations. |

| **RoB 2: Nenti et al. (2025)** [**(5)**](https://sciwheel.com/work/citation?ids=18886967&pre=&suf=&sa=0) | | |
| --- | --- | --- |
| **Bias** | **Authors’ judgement** | **Risk of bias judgement** |
| Domain 1: Risk of bias arising from the randomization process | Low risk of bias | The parent HISTORIC study used randomized treatment allocation, and no evidence of baseline imbalance or problems arising from randomization was identified. Although detailed information regarding allocation concealment was not provided in the pharmacogenetic report, the randomized, double-blind design supports a low risk of bias. |
| Domain 2: Risk of bias due to deviations from the intended interventions (*effect of assignment to intervention*) | Low risk of bias | The randomized intervention was maintained independently of genotype, and the parent trial was double-blinded. The statistical analyses appropriately evaluated treatment effects according to randomized groups. The pharmacogenetic nature of the analysis does not introduce concerns regarding deviations from assigned intervention. |
| Domain 3: Missing outcome data | Low risk of bias | Although the pharmacogenetic analysis was restricted to participants with available DNA samples, the authors explicitly evaluated this limitation and reported that the analysed subset was representative of the overall trial population. Therefore, selection bias due to missing genetic data is unlikely to have materially influenced the findings. |
| Domain 4: Risk of bias in measurement of the outcome | Low risk of bias | Outcome measurement was performed within a blinded randomized clinical trial. The evaluated outcomes were objective or validated clinical measures, reducing the possibility that knowledge of treatment allocation influenced assessment. |
| Domain 5: Risk of bias in selection of the reported result | Some concerns | The main limitation relates to the exploratory nature of the pharmacogenetic analysis. Multiple clinical outcomes and genetic models were evaluated, increasing analytical flexibility. However, the study applied statistical corrections for multiple comparisons and performed Hardy–Weinberg equilibrium testing and genotype quality assessment. |
| Other bias | None to report | None to report |
| Overall risk of bias | Low risk of bias with some concerns regarding selective reporting | The study is based on a well-designed randomized, placebo-controlled, double-blind trial, providing protection against bias related to treatment allocation, deviations from intervention and outcome measurement. The main limitation arises from the secondary pharmacogenetic nature of the analysis, including exploration of multiple genetic models and outcomes. However, the genetic subset was representative of the parent trial population and appropriate statistical approaches were applied, supporting an overall low risk of bias. |

| **RoB 2: Hosking et al. (2021)** [**(6)**](https://sciwheel.com/work/citation?ids=18886972&pre=&suf=&sa=0) | | |
| --- | --- | --- |
| **Bias** | **Authors’ judgement** | **Risk of bias judgement** |
| Domain 1: Risk of bias arising from the randomization process | Low risk of bias | The genetic analysis was nested within a well-established randomized, double-blind trial, and no evidence of imbalance between treatment groups was reported. |
| Domain 2: Risk of bias due to deviations from the intended interventions (*effect of assignment to intervention*) | Low risk of bias | Treatment allocation was randomized and blinded, and the pharmacogenetic analyses were performed according to the assigned treatment groups. No important deviations from the intended interventions were identified. |
| Domain 3: Missing outcome data | Low risk of bias | Although the analysis included only participants with available genetic information, there was no evidence that missing genetic data were related to clinical outcomes. Genotyping quality control procedures reduced the possibility of selection bias. |
| Domain 4: Risk of bias in measurement of the outcome | Low risk of bias | Outcome measurements were obtained within a randomized and blinded clinical trial. The assessed clinical endpoints were standardized and unlikely to be influenced by knowledge of treatment allocation. |
| Domain 5: Risk of bias in selection of the reported result | Some concerns | The main limitation is the exploratory nature of the pharmacogenetic analysis, with multiple variants, treatment groups, endpoints and subgroup analyses evaluated. Although correction for multiple genetic testing was applied, the possibility of selective reporting cannot be completely excluded |
| Other bias | None to report | None to report |
| Overall risk of bias | Low risk of bias with some concerns | The study was based on a large randomized, double-blind clinical trial with appropriate treatment allocation and standardized outcome assessment. The main concerns relate to the secondary pharmacogenetic design and the multiple genetic and clinical analyses performed. However, these limitations are partially mitigated by genetic quality control procedures and adjustment for population structure, supporting an overall low risk of bias. |

| **RoB 2: Condreay et al. (2019)** [**(7)**](https://sciwheel.com/work/citation?ids=14730691&pre=&suf=&sa=0) | | |
| --- | --- | --- |
| **Bias** | **Authors’ judgement** | **Risk of bias judgement** |
| Domain 1: Risk of bias arising from the randomization process | Low risk of bias | The study included participants derived from randomized clinical trials, and no evidence of baseline differences suggesting problems with the randomization process was reported. Although detailed information regarding the randomization procedures of the contributing trials was not provided, the available information does not indicate concerns related to the randomization process. |
| Domain 2: Risk of bias due to deviations from the intended interventions (*effect of assignment to intervention*) | Some concerns | The pharmacogenetic analysis was derived from randomized clinical trials; however, limited information was available regarding blinding, treatment adherence, and protocol deviations across the contributing studies. Although the genetic analyses were appropriately conducted according to FSC exposure, uncertainties regarding the underlying trials prevent a fully low-risk judgement. |
| Domain 3: Missing outcome data | Some concerns | The analysis included only participants with available genetic samples and successful genotyping, representing a subset of the original ITT population. Although the authors reported comparability between the genetic analysis population and the full trial population, selection bias due to availability of genetic material cannot be completely excluded. |
| Domain 4: Risk of bias in measurement of the outcome | Low risk of bias | The outcomes analysed were validated COPD clinical measures collected within clinical trials. Although information on blinding of outcome assessors was limited, the objective nature and standardized assessment of these outcomes reduce the likelihood of measurement bias. |
| Domain 5: Risk of bias in selection of the reported result | Some concerns | The main concern relates to the exploratory nature of the pharmacogenetic analysis. Multiple genetic variants and clinical endpoints were assessed, creating potential analytical flexibility. Although genome-wide significance thresholds were applied, the absence of a prespecified analysis plan limits certainty regarding selective reporting. |
| Other bias | None to report | None to report |
| Overall risk of bias | Some concerns | This study benefits from being derived from randomized clinical trials and from the use of standardized COPD efficacy outcomes. However, some concerns remain due to its secondary post hoc pharmacogenetic design, the restriction of analyses to participants with available genetic samples, and the evaluation of multiple genetic variants and clinical endpoints. Although the genetic analysis population was comparable with the original ITT population, and no evidence of problems related to randomization was identified, the exploratory nature of the study prevents a judgement of low risk of bias overall. |

[Bibliography](https://sciwheel.com/work/bibliography)

[1.	Condreay L, Huang L, Harris E, Brooks J, Riley JH, Church A, et al. Genetic effects on treatment response of umeclidinium/vilanterol in chronic obstructive pulmonary disease. Respir Med. 2016 May;114:123–6.](https://sciwheel.com/work/bibliography/18892976)

[2.	Dmytriiev K, Mostovoy Y, Slepchenko N, Smereka Y. Clinical course of COPD in patients with Arg16Gly (rs1042713) polymorphism of ADRB2 gene. Monaldi Arch Chest Dis. 2022 Sep 13;93(2).](https://sciwheel.com/work/bibliography/18886971)

[3.	Karimi L, Lahousse L, Ghanbari M, Terzikhan N, Uitterlinden AG, van der Lei J, et al. β2-Adrenergic Receptor (ADRB2) Gene Polymorphisms and Risk of COPD Exacerbations: The Rotterdam Study. J Clin Med. 2019 Nov 1;8(11).](https://sciwheel.com/work/bibliography/19023379)

[4.	Dmytriiev K, Mostovoy Y, Slepchenko N, Dmytriiev D, Nazarchuk O, Smereka Y. Does the Gln27Gln polymorphism of the adrenoreceptor beta 2 gene affect the clinical course of chronic obstructive pulmonary disease? Biomed Res Ther. 2022 May 31;9(5):5043–50.](https://sciwheel.com/work/bibliography/19033674)

[5.	Ntenti C, Papakonstantinou E, Grize L, Pascarella M, Frye BC, Fähndrich S, et al. SUMF1 Common Variant rs793391 Is Associated with Response to Inhaled Corticosteroids in Patients with COPD. Int J Mol Sci. 2025 Oct 21;26(20).](https://sciwheel.com/work/bibliography/18886967)

[6.	Hosking L, Yeo A, Hoffman J, Chiano M, Fraser D, Ghosh S, et al. Genetics plays a limited role in predicting chronic obstructive pulmonary disease treatment response and exacerbation. Respir Med. 2021 Oct;187:106573.](https://sciwheel.com/work/bibliography/18886972)

[7.	Condreay LD, Qu XA, Anderson J, Compton C, Ghosh S. Genetic effects on efficacy to fluticasone propionate/salmeterol treatment in COPD. Respir Med. 2019 Aug;155:51–3.](https://sciwheel.com/work/bibliography/14730691)

[8.	Masood O, Devi A, Masood S, Kant S, Banerjee M. Association of the CCL2 A/G (rs3760399) gene variant and its expression with COPD risk and response to combined budesonide/formoterol therapy. Egypt J Bronchol. 2026 Dec;20(1):12.](https://sciwheel.com/work/bibliography/19066615)
